# IMPORTANCE OF MULTIPLE CRITERIA FOR PRIORITY SETTING OF HIV/AIDS INTERVENTIONS

**DOI:** 10.1017/S0266462316000039

**Published:** 2015

**Authors:** Noor Tromp, Rozar Prawiranegara, Adiatma Siregar, Deni Sunjaya, Rob Baltussen

**Affiliations:** Radboud Institute of Health Sciences, Radboud University Medical CenterNoor.Tromp@radboudumc.nl; TB-HIV Research Center, Faculty of Medicine, Padjadjaran University; Faculty of Economics and Business, Padjadjaran University; Department of Public Health, Faculty of Medicine, Padjadjaran University; Radboud Institute of Health Sciences, Radboud University Medical Center

**Keywords:** Priority setting, Multi criteria decision making, Indonesia, HIV/AIDS

## Abstract

**Objectives:** This study describes the views of various stakeholders on the importance of different criteria for priority setting of HIV/AIDS interventions in Indonesia.

**Methods:** Based on a general list of criteria and a focus group discussion with stakeholders (*n* = 6), a list was developed of thirty-two criteria that play a role in priority setting in HIV/AIDS control in West-Java province. Criteria were categorized according to the World Health Organization's health system goals and building block frameworks. People living with HIV/AIDS (*n* = 49), healthcare workers (HCW) (*n* = 41), the general population (*n* = 43), and policy makers (*n* = 22) rated the importance of thirty-two criteria on a 5-point Likert-scale. Thereafter, respondents ranked the highest rated criteria to express more detailed preferences.

**Results:** Stakeholders valued the following criteria as most important for the priority setting of HIV/AIDS interventions: an intervention's impact on the HIV/AIDS epidemic, reduction of stigma, quality of care, effectiveness on individual level, and feasibility in terms of current capacity of the health system (i.e., HCW, product, information, and service requirements), financial sustainability, and acceptance by donors. Overall, stakeholders’ preferences for the importance of criteria are similar.

**Conclusions**: Our study design outlines an approach for other settings to identify which criteria are important for priority setting of health interventions. For Indonesia, these study results may be used in priority setting processes for HIV/AIDS control and may contribute to more transparent and systematic allocation of resources.

In Indonesia, priority setting questions have arisen in the context of HIV/AIDS control as it faces one of Asia's fastest growing HIV/AIDS epidemics and resources are scarce. In 2013, an estimated 610,000 people were living with HIV/AIDS (PLWHA) and it is estimated that this number will increase to 1,500,000 by 2020 if the right measures are not taken (1;2). While the government seems to have the epidemic among people who inject drugs (PWID) under control, the prevalence is increasing among female sex workers (FSW) and their clients, men having sex with men (MSM), and the general population. The budget for HIV/AIDS control is far from sufficient; in 2010, only US$ 69 million was spent on HIV/AIDS, while US$ 152 million was estimated to be needed (3;4).

Both issues urge for a wise choice between HIV/AIDS interventions and allocation of resources. Based on the National AIDS Spending Assessment, most resources on national level were spent on curative services (36 percent compared with 28 percent on preventive services) in 2012 (5). At provincial and district level, the allocation of domestic resources is poorly reported and the process of priority setting of interventions could be improved (6). The AIDS commissions (established at national, provincial and district level) is challenged to coordinate the HIV/AIDS response among multiple stakeholders. It aims to develop strategic plans to guide the local planning board on how to allocate the local budget among different government offices. However, the involvement of stakeholders opinion in the strategic planning process could be improved. Also, while various criteria seemed to guide the HIV/AIDS priorities in Indonesia (for example, the impact of interventions on the HIV/AIDS epidemic, adherence to national guidelines and cultural and political acceptability) these remain implicit. Systematic analysis of how different stakeholders value the importance of various criteria could contribute to more systematic, transparent, and accountable priority setting of the HIV/AIDS response and thereby improve the allocation of resources.

Most methods that were introduced to guide resource allocation decisions in health (that is, evidence-based medicine, cost-effectiveness analysis, burden of disease, and equity analysis, rely on one single criteria (mostly cost and cost-effectiveness), while in reality many criteria can play a role (for example, feasibility, equity, cultural, and political factors) (7). Therefore, multi-criteria decision analysis (MCDA) is put forward as one of the most important methods for priority setting, and it provides a systematic process for clarifying what is being taken into account (the “criteria”), how each of those criteria should be measured, and how much importance (“weight”) to put on each (7). It has been successful in various case studies, for example in Ghana, Nepal, and Thailand, where it contributed to transparent and accountable policy making and brought a step forward in rational decision making. However, only a few empirical priority setting studies have included the views of different stakeholders, such as patients and the general population, besides those of policy makers (8;9).

In HIV/AIDS field, the recognition of multiple criteria has risen and is reflected in the WHO programmatic guidance for antiretroviral therapy (ART) that recommends taking besides health impact also equity and feasibility criteria into consideration (10). However, only a few studies have tried to measure explicitly the importance of multiple criteria for HIV/AIDS priority setting (11;12) and worldwide, the main focus remains on how to reduce new infections and AIDS related death. Against this background, this study aims to describe the views of multiple stakeholders on the importance of various criteria for priority setting in HIV/AIDS control in Indonesia.

## METHODS

### Methods for MCDA: Identification of Criteria

In MCDA, criteria can be identified by various approaches (9), for example, a literature study, focus groups discussion with relevant stakeholders, or using more structured approaches such as Q methodology that combines qualitative and quantitative analyses.

In our study, we started with a general list of criteria draft, that was based on the WHO's health system goals and building block frameworks and is published elsewhere (13). We argue that the reasons why stakeholders prioritize certain health interventions is reflected in these two frameworks and that it can be used to categorize criteria. The health system goals framework contains criteria related to five criteria categories: improvement of the level and distribution of health, responsiveness, social & financial risk protection and improved efficiency. The second framework, the health system building blocks, reflects criteria related to the feasibility of criteria and comprises of six categories, that is, service delivery, health workforce, information, medical products vaccines and technology, financing, and leadership/governance (14).

We adapted the draft general list of criteria to the West-Java HIV/AIDS context based on HIV/AIDS priority setting literature (11;15). In addition, a focus group discussion with a lay person, public health expert, healthcare worker, economists, psychologist, and anthropologist working in HIV/AIDS field was conducted. We asked the participants whether any criteria was missing from the list and whether criteria were defined clearly and in line with the Indonesian context. For the discussion, no systematic method was used. In the end, thirty-two criteria remained and these are presented in Table 1. The definitions of the criteria can be found in Supplementary Table 1. Among others, specific HIV/AIDS criteria that were added include “prevention versus treatment,” “HIV risk of target population,” “reduction of stigma in society,” and “marital status” as unmarried people in Indonesia might be more vulnerable as they have less access to reproductive health services.

**Table 1.** Selected Criteria for HIV/AIDS Priority Setting in Indonesia, Categorized According to WHO Health Systems Frameworks

Category		Criteria
**Health system goals**		
Health impact		Individual effectiveness, Safety, Reducing spread of HIV, Prevention or treatment
Health distribution		Income class, Area of living, Sex and gender, Religion, Marital status, Age, Stigmatized groups, Sexual orientation, Responsible or bad luck, Severity of disease, Level of at risk for HIV infection, People who are easy to reach
Responsiveness		Quality of care, Stigma reduction in society
Social & financial protection		Economic Impact
Efficiency		–
**Health system building blocks**		
Service delivery		Service requirements
Health workforce		Health care personnel requirements
Information		Information system requirements
Medical products, vaccines & technologies		Medical products and technology requirements
Financing		In line with previous spending pattern, Unit costs, Budget impact, Sustainability
Leadership/ governance		Political acceptability, Donor acceptability, Cultural acceptability, Religious acceptability, Legal regulations

None of the criteria from the general list were excluded. However, at the time of this study the general list was still in development and the criterion “burden of disease” was not yet part of it and is, therefore, not included in this study.

### Methods for MCDA: Assessing the Importance of Criteria

The importance (also called weights) of criteria can be identified by well-established economic methods like discrete choice experiments and conjoint analysis to uncover participants’ preferences about the importance of the various attributes (criteria) through their choices. Discrete choice experiments have been applied in several MCDA studies but have been criticized for being too technocratic and not being able to include more than six to eight criteria. Simpler and limited applied methods are rating and scaling of criteria and its feasibility for MCDA will, therefore, be explored in this study.

### Study Setting

Our study was embedded in the IMPACT project, a 5-year EU funded project (2006–11), that aimed to respond to the HIV/AIDS problems in Bandung city and West-Java province. West-Java province is one of the worse hit provinces in Indonesia with an estimated number of people living with HIV/AIDS of 59,000 in 2013 (Reukers et al. unpublished data, 2014). The project set up HIV/AIDS services in hospitals, community and society and conducts scientific clinical, epidemiologic, and economic research and has built up strong links with government institutes and civil society. Bandung is the center for HIV/AIDS control as it houses West-Java's governmental referral hospital (Rumah Sakit Hasan Sadikin) with a HIV/AIDS clinic (clinic Teratai) treating over 1,000 patients per year. West-Java has established a range of HIV/AIDS activities, that is, harm reduction interventions for PWID, including methadone maintenance treatment clinics in six cities, voluntary counseling and testing and antiretroviral treatment at hospital and community clinics including outreach activities, and school-based education on sexual- and drug-related risk behavior.

### Data Collection

Our Questionnaire consisted of five parts and is included in the Supplementary Materials. In part A, we asked the general characteristics of the respondent; in part B we presented and explained all thirty-two criteria for HIV/AIDS priority setting and asked the respondent to compare them simultaneously and to rate the importance of each criteria on a 5-point Likert scale, where 1 = “not important at all,” 2 = “important,” 3 = “indifferent,” 4 = “important,” and 5 = “very important.” For some criteria, we asked an additional question to find out the preference for the level of a criteria, for example, whether the respondent has a preference for prevention over treatment, or men over women for the gender criterion. In part C, the respondent was asked to rank the ten most important criteria, based on the highest scores on the Likert-scale. A white board was used with magnets with names of the criteria. If less than ten criteria scored 5 on the Likert-scale, additional criteria were selected that scored four to include ten criteria for the ranking exercise. In part D, the respondents were asked to compare simultaneously eight interventions that each targets only one specific risk groups and to rate the importance on a 5-point Likert-scale (similar to the scale in Part B). A sheet with an overview of the eight risk groups was used. In part E, the respondent could mention any additional criteria for HIV/AIDS priority setting that were missing in the Supplementary Questionnaire. The Supplementary Questionnaire was translated in Indonesian language and Indonesian researchers (R.P., A.S.) tested the face-validity, by checking whether the Indonesian terms reflected the criteria concepts. Four economic bachelor students who received an incentive per interview conducted the interviews and were trained in several sessions to make them familiar with the topic of priority setting and the Supplementary Questionnaire. Thereafter, the questionnaire was piloted tested several times among fifteen respondents to find optimal phrasing of the questions.

The interviewers approached the participants to see if they would like to participate in a face to face interview. The interviews were taken in a quiet place apart from any other people that could possibly influence the results. Subsequently, the interviewers explained the research and topic of priority setting using an information sheet and asked the respondent for informed consent. The duration of the interviews was on average 30 min and the respondent received a souvenir afterward. Data were collected for a 5-month period (May 12, – October 13, 2011).

### Participants

We included four stakeholder groups in our study, that is, policy makers, the healthcare workers, people living with HIV/AIDS (PLWHA), and the general population, on the basis of the importance of their view in decision making. The interviewers contacted through phone some fifty policy makers that were members of the AIDS commissions and 22 agreed to participate and a face to face appointment was made. All were involved in HIV strategic planning and were permanent staff of the AIDS commissions, or representatives of the health office and government planning board (BAPPEDA), either for Bandung city or West-Java province level.

We approached, through telephone, some fifty healthcare workers that were participants in a training on voluntary counseling and testing at the Medical Faculty of Universitas Padjadjaran in Bandung and forty-one agreed to participate in the interviews and an face to face appointment was made. All of them work with HIV/AIDS patients at in- and out-patients wards of Hasan Sadikin hospital in Bandung city.

We approached some sixty PLWHA that were all waiting in the waiting room of the visitors of the outpatient Teratai clinic and forty-nine agreed to participate. Most of these patients were PWID and their partners. We approached some sixty people from the general population visiting Sunday Market in Bandung city and forty-three of them agreed to participate. These people were approached while they were taking a rest at the market. The general characteristics of each group are presented in Table 2. The research was approved by the Bandung Citizens Ethical Committee and the Padjadjaran University Medical Faculty Ethical Committee.

**Table 2.** General Characteristics of Respondents per Stakeholder Group

	Stakeholder groups					
	Policy makers	People living with	Health care workers	General population		
	(*n* = 22)	HIV/AIDS (*n* = 49)	(*n* = 41)	(*n* = 43)	All respondents (*n* = 155)	
Age, mean years (range)	38.5 (20–64)	31.6 (23–41)	37.3 (22–60)	26.5 (18–57)	32.5	(18–64)
Gender						
Male	14	36	14	21	85	(55%)
Female	8	13	27	22	70	(45%)
Marital status						
Not married	6	22	14	31	73	(47%)
Married	15	26	27	12	80	(52%)
Divorced	1	0	0	0	1	(1%)
Missing	0	1	0	0	1	(1%)
Education						
No education	0	1	0	0	1	1%
Elementary school	0	2	0	3	5	3%
Junior high school	1	2	0	3	6	4%
Senior high school	4	24	3	24	55	36%
College	4	8	17	1	30	19%
University	13	12	21	12	58	37%
Religion						
Islam	21	45	37	39	142	91%
Christen	1	4	2	3	10	7%
Catholic	0	0	2	0	2	1%
Hindu	0	0	0	1	1	1%
Occupation						
Government officer	9	3	–	5	17	11%
Private company employee	0	9	–	11	20	13%
Health care worker						
Doctor	2	0	12	0	14	9%
Nurse	0	0	18	0	18	12%
Case manager/admin	0	0	3	0	3	2%
Pharmacist/analyst	0	0	6	0	6	4%
Entrepreneur/freelancer	2	18	0	2	22	14%
Student	2	1	0	19	22	14%
Housewife	0	6	0	4	10	7%
NGO/social worker	5	8	2	0	15	10%
Other	1	0	0	0	1	1%
Unemployed	1	4	0	2	7	5%
Income (monthly in million IDR (US$)^a^)						
0–1.0 (0–116)	2	17	2	28	54	35%
1.1–3.0 (116–347)	6	19	15	10	50	32%
3.1–5.0 (347–579)	2	3	17	3	25	16%
5.1< (579.1)	10	6	6	2	24	16%
Missing	2	4	1	–	2	1%

a Average exchange rate period May–October 2011: 1USD$ = 8642 IDR.

### Data Analysis

All data were entered in Microsoft Office Excel 2010 and average Likert-scores and standard deviations were calculated using SPSS for the importance rating of thirty-two criteria (part B) and the importance of prioritizing certain risk groups (part D). For the analysis of the ranking exercises, a criterion received ten points when ranked in first place, nine in second place, etcetera, and zero points when ranked below the 10^th^ place. Average ranking scores and standard deviations were calculated for each criterion. For part D, the reasons for prioritizing a risk group were entered in Excel and the frequency was counted accordingly.

## RESULTS

### Likert Scale and Ranking Scores

Table 3 shows the importance of thirty-two criteria based on the mean Likert-scale scores per stakeholder group. While looking at the top 10 of criteria, overall the stakeholders (*n* = 155) expressed a preference for interventions that reduce the spread of HIV and stigma, have high quality of care, are effective on individual level, are feasible in terms of current capacity of the health system (that is, healthcare workers, product, information, and service requirements) and have sustainable financing and are accepted by donors. Criteria related to health distribution (that is, equity and prioritization for certain groups in society) are considered least important. Policy makers expressed a particular preference for interventions that are effective in improving health on individual level while healthcare workers, PLWHA and the general population valued reduction in spread of HIV in society as the most important criterion. Based on the top ten criteria, policy makers and healthcare workers also rated that legal rules acceptability was highly important while the general population also considered unit cost relatively important. In general the views of stakeholders overlapped as the top ten criteria are similar.

**Table 3.** Importance of 32 Criteria for HIV/AIDS Priority Setting as Perceived by Different Sakeholder Groups, Based on Mean Likert Scale Scores

			Policy makers	People living with	Health care workers	General population
All respondents (*n* = 155)			(*n* = 22)	HIV/AIDS (*n* = 49)	(*n* = 41)	(*n* = 43)
			
Rank, criteria	Mean score (SD)		Rank			
1 Reduction spread HIV	4.66	(0.57)	5	1	1	1
2 Stigma reduction	4.55	(0.77)	6	5	3	2
3 Health care workers requirements	4.52	(0.73)	3	4	5	5
4 Quality of care	4.50	(0.78)	9	2	2	9
5 Product and technology requirements	4.48	(0.75)	8	7	8	3
6 Individual effectiveness	4.47	(0.63)	1	3	6	10
7 Sustainable financing	4.46	(0.85)	4	10	4	4
8 Service requirements	4.41	(0.73)	2	9	10	6
9 Information system requirements	4.39	(0.72)	7	6	7	12
10 Donors acceptability	4.24	(0.82)	17	8	15	8
11 Legal rules acceptability	4.22	(0.84)	10	13	9	11
12 Unit cost	4.10	(1.24)	16	12	18	7
13 Side effects	4.09	(0.80)	15	11	17	14
14 Religious acceptability	4.08	(1.03)	14	16	12	13
15 Cultural acceptability	4.08	(0.98)	11	14	13	15
16 Prevention or treatment	3.83	(1.31)	20	15	16	17
17 Economic impact	3.76	(1.29)	12	17	14	19
18 Level at risk individual	3.72	(1.46)	21	19	11	16
19 Political acceptability	3.41	(1.47)	13	22	23	18
20 Severity of disease	3.35	(1.46)	18	20	20	20
21 People easy to target	3.32	(1.23)	19	21	21	21
22 Stigmatized groups	3.30	(1.57)	24	18	19	22
23 Age	3.12	(1.56)	22	23	22	23
24 In line with previous spending pattern	2.86	(1.45)	23	24	26	25
25 Area of living	2.80	(1.56)	25	26	24	24
26 Budget impact	2.44	(1.49)	29	25	29	27
27 Responsibility for health	2.43	(1.52)	27	29	25	26
28 Sexual orientation	2.43	(1.55)	28	27	27	28
29 Marital status	2.25	(1.56)	30	28	28	30
30 Income class	2.14	(1.47)	26	30	30	29
31 Gender	1.48	(1.12)	31	31	31	31
32 Religion	1.06	(0.33)	32	32	32	32

SD, standard deviation.

The results of the ranking exercise did not have a huge effect on the importance of criteria and the detailed results are presented in Supplementary Table 2.

### Preferences for Certain Risk Groups

Table 4 shows the preference among stakeholders for a risk group that a HIV/AIDS intervention targets. Policy makers, PLWHA, and healthcare workers find it most important to target PWID while the general population gives most priority to female sex workers. Policy makers prefer to give least priority for transgender and the other three stakeholders for people at low risk of HIV infection. The following five reasons were mostly given for their choices: (i) level of at risk for HIV, (ii) importance in spread of HIV epidemic, (iii) size of population (infected), (iv) equity considerations (a target group's current access to HIV interventions, socioeconomic status and responsibility for HIV infection), and (v) experienced feasibility/effectiveness of existing interventions, for example whether a group is already effectively targeted. An overview of the reasons given by the stakeholders to prioritize certain risk groups is presented in Supplementary Table 3.

**Table 4.** Priority for Targeting Certain Risk Group Given by Different Stakeholder Groups, Based on 5-Point Likert Scale Scores

				Policy makers		People living with HIV/AIDS		Health care workers		General population	
All respondents (*n* = 155)				(*n* = 22)		(*n* = 49)		(*n* = 41)		(*n* = 43)	
		Mean			Mean		Mean		Mean		Mean
Rank	Risk group	score	(SD)	Rank	score	Rank	score	Rank	score	Rank	score
1	People who inject drugs	4.28	(0.74)	1	4.27	1	4.39	1	4.51	2	3.95
2	Female sex workers	4.20	(0.89)	4	4.09	2	4.20	3	4.27	1	4.19
3	Partners of HIV+ people	4.03	(0.90)	3	4.14	7	3.82	2	4.41	3	3.86
4	Clients of FSW	3.80	(1.09)	2	4.27	3	3.65	4	4.02	4	3.53
5	Prisoners	3.58	(1.19)	5	3.77	6	3.55	5	4.00	5	3.12
6	Men having sex with men	3.47	(1.19)	6	3.41	4	3.63	7	3.71	6	3.09
7	Transgender	3.40	(1.03)	8	3.18	5	3.45	6	3.85	7	3.05
8	People low at risk	2.74	(1.29)	7	3.32	8	2.94	8	2.51	8	2.43

FSW, female sex workers; SD, standard deviation.

### Additional Criteria

Twelve healthcare workers, eleven PLWHA, and four persons from the general population mentioned additional criteria for HIV/AIDS priority setting. However, most were criteria already captured in our Supplementary Questionnaire, examples of interventions or irrational criteria for priority setting. One valid criterion mentioned was the human resources capacity within government institutions, and relates to the governance and leadership category of the feasibility criteria.

## DISCUSSION

This study has described the importance of criteria for priority setting of HIV/AIDS interventions in Indonesia using the perspectives of policy makers, PLWHA, healthcare workers, and the general population. The perceived importance of an intervention's impact on the epidemic can be explained by Indonesia's epidemic, which is still one of the fastest growing in Asia (16). It is also in line with the worldwide preference to reduce new infections and AIDS related deaths, as is reflected in the UNAIDS goals for Asia (i.e., zero new infections, zero new death, and zero discrimination). Indonesia's national and West-Java provincial HIV strategies stress the importance of intervention's impact on the epidemic, however, it is not mentioned as an explicit criterion for priority setting (3;6). Similarly, our respondents rated PWID as the most important target group for interventions with the reason that they are important in the spread of the HIV epidemic. At the time of interview, most new infections were indeed seen among PWID, while now, the epidemic has decreased, and MSM, low-at-risk women, and clients of sex workers are most at risk of HIV infection.

The preference among all stakeholders for interventions that provide good quality of care and are feasible in terms of healthcare workers, service, and information requirements can be explained by Indonesian poor quality of care and health system capacity that has not improved much after decentralization of services from national to district level in 2000 (17–19). Currently, the coverage of most HIV interventions is low (for example, 18 percent coverage for ART) (1) and, although scaling up at community level clinics (*Puskesmas*) is recommended, this is challenging regarding Indonesia's current health system infrastructure related to HIV.

Community healthcare workers still have limited knowledge about HIV. Testing and treatment services are established on a small scale at the community level, and monitoring and referral systems work sub-optimally (20). The preference among stakeholder for interventions that reduce stigma in society can be clarified by the high presence of HIV-related stigma in Indonesia among healthcare workers and in society. Many risk groups and HIV patients in Indonesia face stigma-related barriers for accessing care (21). Their high concern for whether and intervention receives sustainable financing and donor acceptability of interventions can be explained by the high amount of donor funding in Indonesia (5).

All stakeholders valued equity criteria related to people's social background characteristics as least important. This could be related to Indonesia's strong community system in which persons are considered equal and may explain why our respondents do not prefer to prioritize people on the basis of income, gender, and sexual orientation (22). It could be that equity considerations are more important in a generalized epidemic like in South Africa where resources are even tighter and questions on how to balance efficiency and equity considerations are more prominent. In addition, as access to treatment is still low (i.e., 18 percent) in Indonesia, it might not be a stakeholder's first concern to consider inequities but how to provide access for as many people as possible. However, surveys show that inequities exist for other health services in Indonesia and may, therefore, also exist for HIV-related interventions (23). Our respondents did mention various equity-related reasons for targeting a specific group. For example, they considered the socioeconomic status and the vulnerability of the target group. This might indicate that our Supplementary Questionnaire did not measure the concept of equity properly.

The exact resource allocation for HIV/AIDS in West-Java province is unknown and this is part of an on-going research project of the authors of this study. Nevertheless, the results of this study can be an input for the strategic planning process of the West-Java provincial AIDS commission and thereby guide resource allocation. Previous evaluations showed that the following criteria played an important but implicit role in the development of strategic plans: an intervention's impact on the HIV/AIDS epidemic, adherence of priorities to National guidelines, and a mix of cultural and political acceptability considerations.

This study shows that indeed an intervention's impact on the epidemic is considered important among stakeholders. In addition, health system constraints and an intervention's impact on stigma should guide resource allocation decisions. This would mean that interventions that have impact on stigma or strengthen the health system capacity deserve higher priority. On the basis of this study it seems that alignment to higher guidelines seems not important and this seems logical as in a decentralized system the provincial should be free to set priorities. Considerations for equity seem to be less relevant and this may mean that interventions that reduce inequities should have less priority.

This study has several weaknesses. First, the Supplementary Questionnaire was challenging for respondents, as they had to become familiar with thirty-two criteria and compare them simultaneously. Second, the policy makers in our study were mainly implementing donor-funded interventions and do not decide on allocation of budget for HIV control. Third, there may be a risk that they perceive criteria important in line with the current resource allocation pattern. Fourth, we used the WHO health systems frameworks as underlying concepts, and another framework may have led to inclusion of a different set of criteria. Fifth, we may have left out important criteria from the list of thirty-two criteria, as stakeholders mentioned “the size of an intervention's target population” as an important criterion to prioritize target groups and “human resources capacity within government institutions” was considered a missing criterion.

This study also has several strengths. First, we have assessed the importance of a broad set of criteria based on an underlying framework. Second, we have included various stakeholders and were able to compare their preferences. Third, we used a Likert-scale to assess the importance and this is an easy method for stakeholders to indicate their preference.

Our study outlines an approach for other countries that would like to elicit the importance of multiple criteria among different stakeholders groups for priority setting of health interventions for HIV/AIDS control or other disease areas. Priority setting questions can arise either on a micro-level, for example, on how to prioritize risk groups for HIV testing and treatment, or on a macro-level, for example, to prioritize interventions for the long-term HIV/AIDS response. We recommend other countries to make a context-specific list of criteria. Criteria likely differ across disease areas or countries and this was illustrated in our study by the inclusion of “marital status” as a new equity criterion. The stakeholders that are relevant may also differ for different priority setting questions and countries and should be based on a stakeholder analysis.

Multi-criteria decision analysis (MCDA) may be used as a framework to guide priority setting processes (24). After the identification of the most important criteria, interventions options should be defined by the stakeholders and compared on all relevant criteria using a performance matrix. Data should be collected on the performance of all interventions for all relevant criteria. Finally, the performance matrix should be discussed among all relevant stakeholders in a deliberative discussion to make a final decision on which interventions should be prioritized and implemented.

## CONCLUSIONS

This study has described the importance of criteria for priority setting of HIV/AIDS interventions in Indonesia using perspectives of policy makers, PLWHA, healthcare workers, and the general population. Our study design outlines an approach for other settings to identify which criteria are important for priority setting of health interventions within or across disease areas. For Indonesia, these study results may be used in priority setting processes for HIV/AIDS control and may contribute to more transparent and systematic allocation of resources.

## Supplementary material

For supplementary material accompanying this paper visit http://dx.doi.org/10.1017/S0266462316000039. click here to view supplementary material

## CONFLICTS OF INTEREST

All authors declare that they have no conflict of interest.

## References

[1] AIDSdatahub. Indonesia country profile 2014 http://www.aidsdatahub.org/en/country-profiles/indonesia (accessed March 31, 2014).

[2] Indonesian National AIDS Commission. Asian epidemic model estimations 2013. Jakarta: Indonesian National AIDS Commission; 2013.

[3] Indonesian National AIDS Commission. Strategy of the national action plan for HIV/AIDS 2010–2014. Jakarta: Indonesian National AIDS Commission; 2009.

[4] Indonesian National AIDS Commission. UNGASS Republic of Indonesia Country Report on the Follow up to the 35 Declaration of Commitment on HIV/AIDS. Reporting Period 2010–2011. Jakarta: Indonesian National AIDS Commission; 2012.

[5] National AIDS Commission Indonesia. National AIDS Spending Assessment 2011–2012 Indonesa. Jakarta: National AIDS Commission Indonesia; 2013.

[6] Tromp N, Prawiranegara R, Riparev Subhan H, et al. Priority setting in HIV control in West Java Indonesia: An evaluation based on the accountability for reasonableness framework. Health Policy Plan. 2015;30:345–355. 24740708 10.1093/heapol/czu020

[7] Baltussen R, Niessen L. Priority setting of health interventions: The need for multi-criteria decision analysis. Cost Eff Resour Alloc. 2006;4:14. 16923181 10.1186/1478-7547-4-14 PMC1560167

[8] Youngkong S, Kapiriri L, Baltussen R. Setting priorities for health interventions in developing countries: A review of empirical studies. Trop Med Int Health. 2009;14:930–939. 19563479 10.1111/j.1365-3156.2009.02311.x

[9] Marsh K, Lanitis T, Neasham D, Orfanos P, Caro J. Assessing the value of healthcare interventions using multi-criteria decision analysis: A review of the literature. PharmacoEconomics. 2014;32:345–365. 24504851 10.1007/s40273-014-0135-0

[10] World Health Organization. WHO Consultation on the Strategic Use of Antiretrovirals (SUFA). 2nd Expert Panel towards Programmatic Guidance. Geneva: World Health Organization; 2012.

[11] Youngkong S, Baltussen R, Tantivess S, Koolman X, Teerawattananon Y. Criteria for priority setting of HIV/AIDS interventions in Thailand: A discrete choice experiment. BMC Health Serv Res. 2010;10:197. 20609244 10.1186/1472-6963-10-197 PMC2912896

[12] Husain S, Kadir M, Fatmi Z. Resource allocation within the National AIDS Control Program of Pakistan: A qualitative assessment of decision maker's opinions. BMC Health Serv Res. 2007;7:11. 17244371 10.1186/1472-6963-7-11 PMC1784085

[13] Tromp N, Baltussen R. Mapping of multiple criteria for priority setting of health interventions: An aid for decision makers. BMC health services research. BMC Health Serv Res. 2012;12:454. 23234463 10.1186/1472-6963-12-454 PMC3565954

[14] World Health Organization. World Health Organization: Everybody's business: Strengthening health systems to improve health outcomes: WHO's framework for Action. Geneva: World Health Organization; 2007.

[15] Cleary SM, Mooney GH, McIntyre DE. Claims on health care: A decision-making framework for equity, with application to treatment for HIV/AIDS in South Africa. Health Policy Plan. 2010;26:464–470. 21186205 10.1093/heapol/czq081 PMC3199038

[16] UNAIDS. Global report: UNAIDS global report on the global AIDS epidemic 2013. Geneva: UNAIDS; 2013.

[17] Diana A, Hollingworth SA, Marks GC. Quality of physical resources of health facilities in Indonesia: A panel study 1993–2007. Int J Qual Health Care. 2013;25:488–496. 23946293 10.1093/intqhc/mzt057

[18] Heywood P, Choi Y. Health system performance at the district level in Indonesia after decentralization. BMC Int Health Hum Rights. 2010;10:3. 20205724 10.1186/1472-698X-10-3 PMC2839983

[19] Heywood PF, Harahap NP. Human resources for health at the district level in Indonesia: The smoke and mirrors of decentralization. Human Resour Health. 2009;7:6. 10.1186/1478-4491-7-6 PMC2662783 19192269

[20] Indonesian National AIDS Commission. Mid-term review of the national AIDS strategy and action plan 2010–2014. Jakarta: Indonesian National AIDS Commission; 2014.

[21] Sasaki Y, Arifin A, Ali M, Kakimoto K. Willingness to undergo HIV testing among factory workers in Surabaya, Indonesia. AIDS Care. 2011;23:1305–1313. 21547754 10.1080/09540121.2011.555745

[22] Hofstede G. Culture's consequences: Comparing values, behaviors, institutions, and organizations across nations, 2nd ed. Thousand Oaks, CA: Sage Publications; 2001.

[23] Utomo B, Sucahya PK, Utami FR. Priorities and realities: Addressing the rich-poor gaps in health status and service access in Indonesia. Int J Equity Health. 2011;10:47. 22067727 10.1186/1475-9276-10-47 PMC3258219

[24] Baltussen R, Mikkelsen E, Tromp N, et al. Balancing efficiency, equity and feasibility of HIV treatment in South Africa – Development of programmatic guidance. Cost Eff Resour Alloc. 2013;11:26. 24107435 10.1186/1478-7547-11-26 PMC3851565

