# Supplementary material for: IMPORTANCE OF MULTIPLE CRITERIA FOR PRIORITY SETTING OF HIV/AIDS INTERVENTIONS
Source: Int J Technol Assess Health Care. 2015;31(6):390–8. doi: 10.1017/S0266462316000039 (PMC4824960; doi:10.1017/S0266462316000039)
Supplement: Supplementary file 1 [file S0266462316000039sup.zip › S0266462316000039sup001/S0266462316000039sup001.docx]

Questionnaire

group

number

interviewer

Criteria for HIV/AIDS priority setting

West-Java province, Indonesia

IMPACT 2011

University of Padjadjaran, Bandung, Indonesia

Researchers:

Dr. Deni Sunyaya

Noor Tromp, MSc

Rozar Prawiranegara, SPsi

Adiatma Siregar, SE MSc

Dr. Rob Baltussen

**INFORMATION SHEET**

Dear Madame/Sir,

Thank your for participating in our study. We would like to give you a short introduction on the topic of our study and de process of the questionnaires:

**Topic: What is priority setting of HIV/AIDS programs?**

- Because HIV/AIDS budgets in West-Java are insufficient to implement every program, for every target group at full scale, the government (i.e. policymakers) **need choose between HIV/AIDS programs**. This process of selecting one program over another is called **priority setting**.
- There are different methods for priority setting and in our study we use multi criteria decision analysis. With this method you try to look at all the different criteria (also called characteristics) of HIV/AIDS programs.
- These differences in criteria of programs make it difficult for policy makers to choose between programs. For example, certain programs target severely ill people but have low impact on improvement of health of the population. While other programs focus on healthy people with a high impact on health of population. Another difficult choice: certain programs focus on well served areas which is relatively cheap and focus on rich people. While other programs, target underserved areas, which is relatively expensive but focus on poorer people.
- As you can see in table 1 each HIV/AIDS program performs different on criteria.

| Table 1. Performance of HIV/AIDS programs on criteria | | | | | |
| --- | --- | --- | --- | --- | --- |
| **HIV/AIDS programs** | **Criteria** | | | | |
|  | **Health  impact** | **Target area** | **Target**  **poor/ rich** | **Financial protection** | **Ease of**  **Implemen-tation** |
| VCT at rural community level to all risk populations | great | rural | rich | small | easy |
| ART provision at urban hospitals | average | urban | both | great | easy |
| Distribution condoms in brothels among clients of FSW | great | urban | both | small | difficult |
| Clean needles for IDUs in urban areas | great | urban | rich | small | difficult |
| Treatment for opportunistic infections among poor populations | small | urban | poor | great | easy |

- Because everyone has a different opinion about the importance of criteria of programs we would like to ask your opinion about this.

Study protocol;

1. First we will ask you to fill in the inform consent and ask some background questions.
2. In part 1 we will try to find out how important you find certain criteria in the choice of HIV/AIDS programs.
3. In part 2 we will ask you how important you find programs that target certain risk groups for HIV/AIDS.
4. In part 3 we will ask you to rank order the criteria on the whiteboard, by using the magnets. The most important criteria should be placed on top and the least at the bottom.

Be aware:

- That we will ask you about your opinion, so feel free to give them, as there are no false or correct answers.
- This interview is confidentially and anonymous, the results will only be used for scientific purposes.
- The duration of the interview is about 1 hour.

**PART A . BACKGROUND CHARACTERISTICS RESPONDENTS**

|  | Variable | | Answer | |
| --- | --- | --- | --- | --- |
| A1. | Date of birth | | Day______ Month______Year______ | |
| A2. | Gender | | ☐ 1. Men  ☐ 2. Women | |
| A3. | Religion | | ☐ 1. Islam  ☐ 2. Christian  ☐ 3. Catholic | ☐ 4. Hindu  ☐ 5. Buddha  ☐ 6. No religion |
| A4. | Marital status | | ☐ 1. Not married  ☐ 2. Married  ☐ 3. Divorced ☐ 4. Widow | |
| A5. | Education (completed) | | ☐ 1. No education  ☐ 2. Elementary school  ☐ 3. Junior high school  ☐ 4. Senior high school  ☐ 5. Collage  ☐ 6. University  ☐ 7. PhD | |
| A6. | Professional background  a. Profession  b. Location (Institution & department/clinic)  c. Rank *(only for policymakers)*  d. Time of start working in profession  e. Working in HIV/AIDS field  f. Time of start working in HIV/AID field | | ____________________ ____________________  ________Rank  Year______ Month______  ☐ 1. Yes ☐ 2. No  Year______ Month______ | |
| A7. | Monthly income | | ☐ 1. Rp. 0-499,999  ☐ 2. Rp. 500,000 – 999,999  ☐ 3. Rp. 1,000,000 – 1,999,999  ☐ 4. Rp. 2,000,000 – 2,999,999  ☐ 5. Rp. 3,000,000 – 3,999,999  ☐ 6. Rp. 4,000,000 – 4,999,999  ☐ 7. Rp. 5,000,000 – 6,999,999  ☐ 8. Rp. more than 7,000,000 | |
| A8. | Address: | | City:_________ Area________ | |
| A9. | Have you ever heard about HIV/AIDS before? | | ☐ 1. Yes  ☐ 2. No | |
| A10. | Have you ever been tested for HIV? | | ☐ 1. Yes  ☐ 2. No | |
| A11. | Phone number | |  | |
|  |  | |  | |
|  | Filled in by interviewer | | | |
| A12. | Teratai database number *(for patients only)* |  | | |
| A13. | Date of interview |  | | |
| A14. | Time part A (minutes): |  | | |
| A15. | Time part B (minutes): |  | | |
| A16. | Time part C (minutes): |  | | |

**START TIME:_______**

**PART B. IMPORTANCE OF CRITERIA IN THE CHOICE OF HIV/AIDS PROGRAMS**

There are many criteria that can play a role in the choice of HIV/AIDS programs, see overview. Please consider all criteria and answer how important each criterion is, in comparison to the other criteria, in the choice of HIV/AIDS programs.

Respond by choosing for:

**unimportant - of little importance - moderately important - important - very important**

Secondly, specify how much priority you would give to certain programs.
Please respond by choosing for:

**very low priority - low priority - indifferent - high priority - very high priority**

**Remember that the budget for HIV/AIDS is limited and that choices between programs have to be made.**

| **How important are the following criteria in the choice of HIV/AIDS programs:** | |
| --- | --- |
| 1. How much a program improves health of the individual.   of little importance  moderately important  important  very important  unimportant | |
| 1. How much side effects a program has.   of little importance  moderately important  important  very important  unimportant | |
| 1. How much a program reduces the spread  of HIV/AIDS in society.   of little importance  moderately important  important  very important  unimportant | |
| 1. Whether a program is about treatment or prevention.   of little importance  moderately important  important  very important  unimportant | |
|  | *How much priority would you give to programs:* |
|  | A4a. that prevent HIV infection compared to programs  that treat HIV/AIDS patients?  of little importance  moderately important  important  very important  unimportant |

| **How important are the following criteria in the choice of HIV/AIDS programs:** | |
| --- | --- |
| 1. Whether it targets people with certain income classes.   of little importance  moderately important  important  very important  unimportant | |
|  | *How much priority would you give to programs that target:* |
|  | B5a. poor people compared to rich and middle class people?  low  priority  indifferent  high  priority  very high priority  very low priority |
|  | B5b. middle class people compared to poor and rich people?  low  priority  indifferent  high  priority  very high priority  very low priority |
|  | B5c. rich people compared to poor and middle class people?  low  priority  indifferent  high  priority  very high priority  very low priority |
| 1. Whether it targets people in underserved or well served areas.   of little importance  moderately important  important  very important  unimportant | |
|  | *How much priority would you give to programs that target:* |
|  | B6a. underserved areas compared to well served areas?  low  priority  indifferent  high  priority  very high priority  very low priority |
| 1. Whether it targets men or women.   of little importance  moderately important  important  very important  unimportant | |
|  | *How much priority would you give to programs that target:* |
|  | B7a. Men compared to women?  low  priority  indifferent  high  priority  very high priority  very low priority |

| **How important are the following criteria in the choice of HIV/AIDS programs:** | | |
| --- | --- | --- |
| 1. Whether it targets people with certain religion?   of little importance  moderately important  important  very important  unimportant | | |
|  | *How much priority would you give to programs that target:* | |
|  | B8a. Muslims compared to other religions?  low  priority  indifferent  high  priority  very high priority  very low priority | |
|  | B8b. Christians compared to other religions?  low  priority  indifferent  high  priority  very high priority  very low priority | |
|  | B8c. Catholics compared to other religions?  low  priority  indifferent  high  priority  very high priority  very low priority | |
|  | B8d. Buddhists compared to other religions?  low  priority  indifferent  high  priority  very high priority  very low priority | |
|  | B8e. Hindus compared to other religions?  low  priority  indifferent  high  priority  very high priority  very low priority | |
| 1. Whether it targets married or unmarried people?   of little importance  moderately important  important  very important  unimportant | | |
|  | *How much priority would you give to programs that target:* | |
|  | B9a. Married people compared to unmarried people?  low  priority  indifferent  high  priority  very high priority  very low priority | |
| 1. Whether it targets people with certain age?   of little importance  moderately important  important  very important  unimportant | | |
|  | *How much priority would you give to programs that target:* | |
|  | B10a. unborn babies of HIV+ mothers compared to other ages?  low  priority  indifferent  high  priority  very high priority  very low priority | |
| **How important are the following criteria in the choice of HIV/AIDS programs:** | | |
|  | | *How much priority would you give to programs that target:* |
|  | | B10b. children age 0-12 years compared to other ages?  low  priority  indifferent  high  priority  very high priority  very low priority |
|  | | B10c. people age of 12-25 years compared to other ages?  low  priority  indifferent  high  priority  very high priority  very low priority |
|  |  | B10d. people age 25-55 years compared to other ages?  low  priority  indifferent  high  priority  very high priority  very low priority |
|  |  | B10e. people above age above 55 years compared  to other ages?  low  priority  indifferent  high  priority  very high priority  very low priority |
| 1. Whether it gives priority to stigmatized people, who have because of stigma less access.   of little importance  moderately important  important  very important  unimportant | | |
|  | | *How much priority would you give to programs that target:* |
|  | | B11a. people who are stigmatized compared to people who are not?  low  priority  indifferent  high  priority  very high priority  very low priority |
| 1. Whether it gives priority to people with certain sexual orientation?   of little importance  moderately important  important  very important  unimportant | | |
|  | | *How much priority would you give to programs that target:* |
|  | | B12a. gay people compared to waria and heterosexuals?  low  priority  indifferent  high  priority  very high priority  very low priority |
|  | | B12b. waria compared to gay people and heterosexuals?  low  priority  indifferent  high  priority  very high priority  very low priority |

| **How important are the following criteria in the choice of HIV/AIDS programs:** | |
| --- | --- |
|  | *How much priority would you give to programs that target:* |
|  | B12c. heterosexuals compared to waria and gay people?  low  priority  indifferent  high  priority  very high priority  very low priority |
| 1. Whether it targets people that can be hold responsible for   their HIV infection or got it because of bad luck?   of little importance  moderately important  important  very important  unimportant | |
| 1. How severely ill the target group is?   of little importance  moderately important  important  very important  unimportant | |
|  | *How much priority would you give to programs that target:* |
|  | B14a. HIV infected people who are not severely ill compared  to severely ill and life threatened AIDS patients?  low  priority  indifferent  high  priority  very high priority  very low priority |
|  | B14b. AIDS patients who are severely ill compared to HIV infected and life threatened AIDS patients?  low  priority  indifferent  high  priority  very high priority  very low priority |
|  | B14c. Life threatened AIDS patients compared to HIV infected and severely ill AIDS patients?  low  priority  indifferent  high  priority  very high priority  very low priority |
| 1. How much at risk the target group is for HIV infection?   of little importance  moderately important  important  very important  unimportant | |
|  | *How much priority would you give to programs that target:* |
|  | B15a. people who are at high risk for HIV infection  compared to people at low risk?  low  priority  indifferent  high  priority  very high priority  very low priority |
| 1. Whether it has high impact on the economic situation of an area?   of little importance  moderately important  important  very important  unimportant | |
| 1. Whether it targets people who are easy to target?   of little importance  moderately important  important  very important  unimportant | |

| **How important are the following criteria in the choice of HIV/AIDS programs:** |
| --- |
| 1. Whether it satisfies people’s quality of care needs,   e.g. confidentiality, autonomy and dignity.   of little importance  moderately important  important  very important  unimportant |
| 1. Whether it reduces stigma in society.   of little importance  moderately important  important  very important  unimportant |
| 1. Whether it is supported by the already established   service capacity.   of little importance  moderately important  important  very important  unimportant |
| 1. Whether it is supported by the already established   health care workers capacity.   of little importance  moderately important  important  very important  unimportant |
| 1. Whether it is supported by the already established   information system capacity.   of little importance  moderately important  important  very important  unimportant |
| 1. Whether it is supported by the already available   medical products and technology.   of little importance  moderately important  important  very important  unimportant |
| 1. Whether it is in line with previous spending   of little importance  moderately important  important  very important  unimportant |
| 1. Whether it consumes a small part of the government budget.   of little importance  moderately important  important  very important  unimportant |
| 1. Whether it is cheap per patient.   of little importance  moderately important  important  very important  unimportant |
| 1. Whether it has sustainable financing.   of little importance  moderately important  important  very important  unimportant |
| 1. Whether it is political acceptable.   of little importance  moderately important  important  very important  unimportant |

| **How important are the following criteria in the choice of HIV/AIDS programs:** |
| --- |
| 1. Whether it is accepted by international donors.   of little importance  moderately important  important  very important  unimportant |
| 1. Whether it is cultural acceptable.   of little importance  moderately important  important  very important  unimportant |
| 1. Whether it is accepted by religions.   of little importance  moderately important  important  very important  unimportant |
| 1. Whether it is in line with legal regulations.   of little importance  moderately important  important  very important  unimportant |

**END TIME: ______**

**PART C. OTHER CRITERIA THAT PLAY A ROLE IN THE CHOICE OF HIV/AIDS PROGRAMS**

| Are there any other criteria that you find important in the choice of HIV/AIDS programs? | |
| --- | --- |
| *Number* | *Criteria* |
| C1. |  |
| C2. |  |
| C3. |  |
| C4. |  |
| C5. |  |

**START TIME: _____ END TIME: ______**

**START TIME: ________**

**PART D. HIV/AIDS PROGRAMS THAT TARGET SPECIFIC RISK GROUPS**

The following questions focus on how much priority you would give to programs target specific HIV/AIDS risk groups.

Compare the following 8 programs and fill in how much priority you would give each of them:

Program C1: targets **injecting drug users**  Program C5: targets **waria**

Program C2: targets **female sex workers** Program C6: targets **prisoners**

Program C3: targets **clients of female sex workers** Program C7: targets **partners of HIV+ people**

Program C4: targets **gay people** Program C8: targets **people at low HIV risk**

Please choose for:

**very low priority - low priority - indifferent - high priority - very high priority**

In addition, please give your reasons for your answer.

low
priority

indifferent

high
priority

very high priority

very low priority

low
priority

indifferent

high
priority

very high priority

very low priority

| I would give programs that target: |
| --- |
| 1. Injecting drug users |
| Because, compared to other HIV risk groups, injecting drug users: |
| 1. Female sex workers |
| Because, compared to other HIV risk groups, female sex workers: |
| 1. Clients of female sex workers |
| Because, compared to other HIV risk groups, clients of female sex workers: |
| 1. Gay people |
| Because, compared to other HIV risk groups, gay people: |

low
priority

indifferent

high
priority

very high priority

very low priority

low
priority

indifferent

high
priority

very high priority

very low priority

| I would give programs that target: |
| --- |
| 1. Waria   Because, compared to other HIV risk groups, waria: |
| 1. Prisoners |
| Because, compared to other HIV risk groups, prisoners: |
| 1. Partners of HIV+ people |
| Because, compared to other HIV risk groups, partners of HIV positive persons: |
| 1. People at low HIV risk |
| Because, compared to other HIV risk groups, people at low HIV risk: |

low
priority

indifferent

high
priority

very high priority

very low priority

low
priority

indifferent

high
priority

very high priority

very low priority

low
priority

indifferent

high
priority

very high priority

very low priority

low
priority

indifferent

high
priority

very high priority

very low priority

**END TIME: ________**

**START TIME: _____ END TIME: ______**

**PART E. RANKING EXERCISE OF HIV/AIDS CRITERIA**

Please rank the importance of criteria of HIV/AIDS programs on the white board. Put the most important criteria on top and the least important at the bottom.

| Ranking results | | *End of the interview.  Thank you for participation!* |
| --- | --- | --- |
| *Rank* | *Criteria numbers* |  |
|  |  |  |
|  |  |  |
|  |  |  |
|  |  |  |
|  |  |  |
|  |  |  |
|  |  |  |
|  |  |  |
|  |  |  |
|  |  |  |
|  |  |  |
|  |  |  |
|  |  |  |
|  |  |  |
|  |  |  |
|  |  |  |
|  |  |  |
|  |  |  |
|  |  |  |
|  |  |  |
|  |  |  |
|  |  |  |
|  |  |  |
|  |  |  |
|  |  |  |
|  |  |  |
|  |  |  |
|  |  |  |
|  |  |  |
|  |  |  |
|  |  |  |
|  |  |  |
|  |  |  |
|  |  |  |
|  |  |  |
|  |  |  |
