# Supplementary material for: IMPORTANCE OF MULTIPLE CRITERIA FOR PRIORITY SETTING OF HIV/AIDS INTERVENTIONS
Source: Int J Technol Assess Health Care. 2015;31(6):390–8. doi: 10.1017/S0266462316000039 (PMC4824960; doi:10.1017/S0266462316000039)
Supplement: Supplementary file 1 [file S0266462316000039sup.zip › S0266462316000039sup001/S0266462316000039sup002.docx]

| Supplementary table 1. Selected criteria and their definitions for HIV/AIDS priority setting in Indonesia, categorized according to WHO health systems frameworks. | | | | |
| --- | --- | --- | --- | --- |
| Category |  | Criteria |  | Definition |
| **Health system goals** | | | | |
| *Health impact* |  | Individual effectiveness |  | Effect on health, e.g prevention of HIV infection, the extension of life of HIV/AIDS patients |
|  |  | Safety |  | The side effects in terms of health of an intervention |
|  |  | Reducing spread of HIV |  | The impact on de spread of the epidemic. |
|  |  | Prevention or Treatment |  | Prevention: to prevent HIV infection. Treatement: treatment of HIV and AIDs patients |
| *Health distribution* |  | Income class |  | Income of the target group of an intervention |
|  |  | Area of living |  | Area of living of target group. Well served: area’s where people have good access to care, education, living circumstances etc. Underserved, no good access and living circumstances |
|  |  | Sex and gender |  | Gender of target group of intervention. Does it focus on men or women? |
|  |  | Religion |  | Religion of target group of intervention |
|  |  | Marital status |  | Marital status of target group of intervention, married or unmarried men/women. Is not the same as not sexual active and active. |
|  |  | Age |  | Age of target group of intervention |
|  |  | Stigmatized groups |  | Whether an intervenion targets stigmatized groups. Stigmatization is not only related to health and could related to stigmatized persons and/or their family. |
|  |  | Sexual orientation |  | Sexual orientation of target group. Gay people = men having sex with men. Waria = men who want to be a women and act like a women. Biseksual and lesbian is excluded as sexual orientation group. |
|  |  | Responsible or bad luck |  | Whether the target group of an intervention is responsible for his/her HIV infection (client of sex workers, Injecting drug users, etc). In bad luck there is no or less responsibility, e.g. the partners of HIV+ people and children of HIV+ mothers. |
|  |  | Severity of disease |  | How servely ill the target group is. |
|  |  | Level of at risk for HIV infection |  | The level of risk for HIV of the target group, now and in the future. E.g. high risk: injecting drug users, female sex worker. Low risk: children in high school, general population. |
|  |  | People who are  easy to reach |  | Some people are easier to reach with interventions, e.g. partners of HIV+ people and prisoners, compared to men having sex with men, as they are not open about their sexual orientation. |
| *Responsiveness* |  | Quality of care |  | Quality of care of a service. How is a persons treated, is it confidential, can people protest, have they autonomy. |
|  |  | Stigma reduction in society |  | Reduction of stigma opinion in society. So targetting the general public and people who stigmatize. |
| *Social & financial protection* |  | Economic Impact |  | These interventions target people who are in working/productive age. As if they are not targeted they cannot work because of HIV/AIDS and fall into poverty. And protection of people against the cost of illness. This intervention protects then economy in West-Java. |
| **Health system building blocks / Feasibility** | | | | |
| *Service delivery* |  | Service requirements |  | What is the current capacity of services in the health care system and how easy would it be implement the new intervention? What are additional requirements. |
| *Health workforce* |  | Health care personnel requirements |  | What is the current capacity of health care personnel in the health care system and how easy would it be implement the new intervention? What are additional requirements. |
| *Information* |  | Information system requirements |  | What is the current capacity of information system to monitor patient and the service in the health care system and how easy would it be implement the new intervention? What are additional requirements. E.g. is a referral system present, to refer patients from one clinic to the other? |
| *Medical products, vaccines & technologies* |  | Medical products and technology requirements |  | What is the current capacity of medical products and technology in the health care system and how easy would it be implement the new intervention? What are additional requirements. For example is testing material already present? |
| *Financing* |  | In line with previous spending pattern |  | Whether money was spend on this intervention last year. |
|  |  | Unit costs |  | What are the total cost per patient, from a societal perspective. So all costs to run the service, not only the costs for patient. |
|  |  | Budget impact |  | What is the part of the available budget that the intervention will consume. |
|  |  | Sustainability |  | Wheter there is sustainble financing. |
| *Leadership/ governance* |  | Political acceptability |  | Whether the intervention will be accepted by political leades and political influence. |
|  |  | Donor acceptability |  | Whether it is in line with requirements of donors, in order to receive funding for the intervention. |
|  |  | Cultural acceptability |  | Whether the general public will accept this intervention. |
|  |  | Religious acceptability |  | Whether the religions and their leaders will accept this intervention. |
|  |  | Legal regulations |  | Whether the intervention is in line with legal regulations. |
