# Supplementary material for: IMPORTANCE OF MULTIPLE CRITERIA FOR PRIORITY SETTING OF HIV/AIDS INTERVENTIONS
Source: Int J Technol Assess Health Care. 2015;31(6):390–8. doi: 10.1017/S0266462316000039 (PMC4824960; doi:10.1017/S0266462316000039)
Supplement: Supplementary file 1 [file S0266462316000039sup.zip › S0266462316000039sup001/S0266462316000039sup003.docx]

| Supplementary table 2. Mean ranking scores and standard deviation per criterion and per stakeholder group | | | | | | | | | | | | | | | | | | | | | | | | |
| --- | --- | --- | --- | --- | --- | --- | --- | --- | --- | --- | --- | --- | --- | --- | --- | --- | --- | --- | --- | --- | --- | --- | --- | --- |
| Stakeholder groups | | | | | | | | | | | | | | | | | | | | | | | | |
| Policy makers (n=22) | | | |  | People living with HIV/AIDS (n=49) | | | |  | Health care workers (n=41) | | | | |  | General population (n=43) | | | |  | Overall (n=155) | | | |
|  | Criterion | Mean (SD) | |  |  | Criterion | Mean (SD) | |  |  | Criterion | | Mean (SD) | |  |  | Criterion | Mean (SD) | |  |  | Criterion | Mean (SD) | |
| 1 | Reduction spread HIV | 5.23 | (4.29) |  | 1 | Reduction spread HIV | 5.43 | (4.04) |  | 1 | Reduction spread HIV | 7.24 | | (3.95) |  | 1 | Reduction spread HIV | 5.58 | (4.34) |  | 1 | Reduction spread HIV | 5.92 | (4.17) |
| 2 | Service requirements | 4.50 | (3.54) |  | 2 | Quality of care | 5.00 | (3.22) |  | 2 | Quality of care | 4.71 | | (3.76) |  | 2 | HCWs requirements | 3.95 | (3.30) |  | 2 | Individual effectiveness | 2.41 | (3.17) |
| 3 | Sustainable financing | 4.50 | (3.76) |  | 3 | HCWs requirements | 4.47 | (3.48) |  | 3 | Level at risk individual | 3.85 | | (3.89) |  | 3 | Products requirements | 3.77 | (2.95) |  | 3 | Quality of care | 4.34 | (3.44) |
| 4 | Quality of care | 4.27 | (3.76) |  | 4 | Prevention or treatment | 3.84 | (4.40) |  | 4 | Stigma reduction | 3.76 | | (3.53) |  | 4 | Stigma reduction | 3.72 | (3.77) |  | 4 | HCWs requirements | 4.05 | (3.29) |
| 5 | Stigma reduction | 4.23 | (3.19) |  | 5 | Stigma reduction | 3.80 | (3.84) |  | 5 | Prevention or treatment | 3.66 | | (4.14) |  | 5 | Prevention or treatment | 3.26 | (4.24) |  | 5 | Stigma reduction | 3.83 | (3.62) |
| 6 | HCWs requirements | 4.14 | (3.11) |  | 6 | Unit cost | 3.24 | (3.56) |  | 6 | HCWs requirements | 3.61 | | (3.20) |  | 6 | Quality of care | 3.26 | (3.05) |  | 6 | Prevention or treatment | 3.36 | (4.18) |
| 7 | Individual effectiveness | 3.68 | (3.43) |  | 7 | Information requirements | 3.22 | (3.51) |  | 7 | Sustainable financing | | 3.22 | (3.63) |  | 7 | Level at risk individual | 2.98 | (4.08) |  | 7 | Service requirements | 3.06 | (3.25) |
| 8 | Information requirements | 2.45 | (3.28) |  | 8 | Service requirements | 2.98 | (3.24) |  | 8 | Service requirements | | 3.05 | (3.37) |  | 8 | Individual effectiveness | 2.58 | (3.54) |  | 8 | Products requirements | 2.85 | (2.90) |
| 9 | Political acceptability | 2.45 | (3.96) |  | 9 | Products requirements | 2.82 | (2.71) |  | 9 | Information requirements | | 2.59 | (2.83) |  | 9 | Unit cost | 2.44 | (3.15) |  | 9 | Sustainable financing | 2.72 | (3.32) |
| 10 | Legal rules acceptability | 2.36 | (3.58) |  | 10 | Individual effectiveness | 2.22 | (2.92) |  | 10 | Products requirements | | 2.24 | (2.89) |  | 10 | Service requirements | 2.42 | (2.86) |  | 10 | Information requirements | 2.59 | (3.21) |
| 11 | Products requirements | 2.27 | (2.95) |  | 11 | Donors acceptability | 2.08 | (3.22) |  | 11 | Individual effectiveness | | 1.78 | (2.80) |  | 11 | Sustainable financing | 2.19 | (2.99) |  | 11 | Level at risk individual | 2.51 | (3.65) |
| 12 | Religious acceptability | 2.18 | (3.76) |  | 12 | Sustainable financing | 1.98 | (2.81) |  | 12 | Stigmatized groups | | 1.68 | (2.48) |  | 12 | Information requirements | 1.95 | (3.15) |  | 12 | Unit cost | 2.30 | (3.15) |
| 13 | Prevention or treatment | 1.95 | (3.48) |  | 13 | Level at risk individual | 1.65 | (3.06) |  | 13 | Economic impact | | 1.59 | (2.86) |  | 13 | Side effects | 1.81 | (3.06) |  | 13 | Religious acceptability | 1.43 | (2.81) |
| 14 | Economic impact | 1.82 | (2.48) |  | 14 | Side effects | 1.37 | (2.51) |  | 14 | Unit cost | | 1.41 | (2.48) |  | 14 | Severity of disease | 1.77 | (3.31) |  | 14 | Economic impact | 1.42 | (2.60) |
| 15 | Cultural acceptability | 1.73 | (3.17) |  | 15 | Religious acceptability | 1.37 | (2.77) |  | 15 | Side effects | | 1.39 | (2.75) |  | 15 | Legal rules acceptability | 1.72 | (2.76) |  | 15 | Side effects | 1.41 | (2.68) |
| 16 | Unit cost | 1.55 | (2.82) |  | 16 | Economic impact | 1.35 | (2.39) |  | 16 | Severity of disease | | 1.22 | (2.29) |  | 16 | Religious acceptability | 1.49 | (2.88) |  | 16 | Donors acceptability | 1.39 | (2.63) |
| 17 | Level at risk individual | 1.00 | (2.43) |  | 17 | Stigmatized groups | 1.20 | (2.92) |  | 17 | Religious acceptability | | 1.02 | (2.14) |  | 17 | Age | 1.42 | (2.78) |  | 17 | Legal rules acceptability | 1.30 | (2.64) |
| 18 | Severity of disease | 0.77 | (1.66) |  | 18 | Severity of disease | 1.12 | (2.65) |  | 18 | Donors acceptability | | 0.98 | (2.33) |  | 18 | Donors acceptability | 1.35 | (2.36) |  | 18 | Severity of disease | 1.28 | (2.65) |
| 19 | Side effects | 0.73 | (2.10) |  | 19 | Political acceptability | 1.06 | (2.66) |  | 19 | Legal rules acceptability | | 0.88 | (2.48) |  | 19 | Area of living | 1.26 | (2.83) |  | 19 | Stigmatized groups | 1.14 | (2.45) |
| 20 | Donors acceptability | 0.73 | (1.91) |  | 20 | Age | 0.82 | (2.21) |  | 20 | Age | | 0.76 | (1.96) |  | 20 | Economic impact | 1.14 | (2.70) |  | 20 | Political acceptability | 0.99 | (2.49) |
| 21 | Stigmatized groups | 0.55 | (1.50) |  | 21 | Legal rules acceptability | 0.82 | (1.98) |  | 21 | Political acceptability | | 0.71 | (1.90) |  | 21 | Stigmatized groups | 0.86 | (2.18) |  | 21 | Age | 0.90 | (2.24) |
| 22 | Previous spending | 0.55 | (1.87) |  | 22 | Cultural acceptability | 0.76 | (2.12) |  | 22 | Cultural acceptability | | 0.63 | (1.53) |  | 22 | Cultural acceptability | 0.77 | (2.02) |  | 22 | Cultural acceptability | 0.86 | (2.15) |
| 23 | Income class | 0.41 | (1.22) |  | 23 | Budget impact | 0.63 | (2.05) |  | 23 | Sexual orientation | | 0.51 | (1.86) |  | 23 | Marital status | 0.60 | (1.69) |  | 23 | Area of living | 0.52 | (1.83) |
| 24 | Age | 0.36 | (1.33) |  | 24 | Sexual orientation | 0.39 | (1.34) |  | 24 | Easy to target | | 0.46 | (1.25) |  | 24 | Easy to target | 0.56 | (1.71) |  | 24 | Easy to target | 0.43 | (1.39) |
| 25 | Sexual orientation | 0.18 | (0.85) |  | 25 | Easy to target | 0.39 | (1.44) |  | 25 | Marital status | | 0.44 | (1.29) |  | 25 | Responsibility for health | 0.49 | (1.65) |  | 25 | Sexual orientation | 0.41 | (1.56) |
| 26 | Easy to target | 0.18 | (0.66) |  | 26 | Marital status | 0.27 | (1.06) |  | 26 | Area of living | | 0.39 | (1.41) |  | 26 | Sexual orientation | 0.44 | (1.78) |  | 26 | Marital status | 0.37 | (1.26) |
| 27 | Responsibility for health | 0.14 | (0.64) |  | 27 | Responsibility for health | 0.18 | (0.95) |  | 27 | Gender | | 0.39 | (1.76) |  | 27 | Political acceptability | 0.44 | (1.33) |  | 27 | Budget impact | 0.32 | (1.46) |
| 28 | Area of living | 0.09 | (0.29) |  | 28 | Previous spending | 0.18 | (0.83) |  | 28 | Budget impact | | 0.37 | (1.64) |  | 28 | Gender | 0.33 | (1.38) |  | 28 | Responsibility for health | 0.25 | (1.11) |
| 29 | Gender | 0.00 | (0.00) |  | 29 | Area of living | 0.16 | (1.14) |  | 29 | Income class | | 0.20 | (1.10) |  | 29 | Income class | 0.23 | (1.15) |  | 29 | Gender | 0.23 | (1.19) |
| 30 | Religion | 0.00 | (0.00) |  | 30 | Income class | 0.10 | (0.51) |  | 30 | Responsibility for health | | 0.15 | (0.69) |  | 30 | Previous spending | 0.14 | (0.91) |  | 30 | Income class | 0.21 | (0.98) |
| 31 | Marital status | 0.00 | (0.00) |  | 31 | Gender | 0.10 | (0.51) |  | 31 | Previous spending | | 0.12 | (0.78) |  | 31 | Budget impact | 0.07 | (0.46) |  | 31 | Previous spending | 0.21 | (1.05) |
| 32 | Budget impact | 0.00 | (0.00) |  | 32 | Religion | 0.00 | (0.00) |  | 32 | Religion | | 0.00 | (0.00) |  | 32 | Religion | 0.00 | (0.00) |  | 32 | Religion | 0.00 | (0.00) |
| ^a^ HCWs = health care workers, ^b^ products = medical products & technology requirements, ^c^ information = information system requirements, ^d^ previous spending = in line with previous spending patterns | | | | | | | | | | | | | | | | | | | | | | | | |
