# Supplementary material for: IMPORTANCE OF MULTIPLE CRITERIA FOR PRIORITY SETTING OF HIV/AIDS INTERVENTIONS
Source: Int J Technol Assess Health Care. 2015;31(6):390–8. doi: 10.1017/S0266462316000039 (PMC4824960; doi:10.1017/S0266462316000039)
Supplement: Supplementary file 1 [file S0266462316000039sup.zip › S0266462316000039sup001/S0266462316000039sup004.docx]

| Supplementary table 3. Reasons and frequency given by stakeholders to prioritize or not prioritize certain risk groups in HIV/AIDS control | | | | | | | | | | | | | | | | | |
| --- | --- | --- | --- | --- | --- | --- | --- | --- | --- | --- | --- | --- | --- | --- | --- | --- | --- |
|  | |  | Reasons to prioritize risk group (Likert scores 3-5) | | | | | |  | Reasons to not prioritize group (Likert scores 1-3) | | | | | | | |
| Risk groups and  stakeholders  (# missing reasons) | |  | At high  HIV risk | Important in spread of HIV epidemic | Large number of people (infected) | Equity: less access to services, more vulnerable | Feasibility: effective interventions are available/ easy group to reach | Feasibility: no effective interventions available/ difficult group to target | Other | At low HIV risk | Not important in spread of HIV epidemic | Small number of people (infected) | Equity: group with relatively high socio economic status | Equity: own responsibility | Feasibility: effective interventions are available/ group is already targeted | Feasibility: no effective interventions available/ difficult group to target | No need for services (already show safe behaviour) |
| **IDUs** | |  |  |  |  |  |  |  |  |  |  |  |  |  |  |  |  |
| Policy makers (1) | |  | 3 | 10 | 5 |  | 1 | 3 |  |  |  |  |  |  |  |  |  |
| Health care workers (1) | |  | 16 | 15 | 12 |  |  | 1 |  |  |  |  |  |  | 2 |  |  |
| PLWHA (1) | |  | 20 | 9 | 4 | 4 |  | 4 | 1 |  |  |  |  | 1 |  | 1 |  |
| General population (0) | |  | 23 | 3 | 5 | 1 |  | 5 |  |  |  | 1 | 1 | 1 |  |  | 3 |
| **FSWs** | |  |  |  |  |  |  |  |  |  |  |  |  |  |  |  |  |
| Policy makers (1) | |  | 3 | 9 |  | 4 | 2 | 1 |  |  | 1 | 1 |  |  |  |  |  |
| Health care workers (2) | |  | 15 | 15 | 1 | 2 |  | 9 |  |  |  |  |  |  | 2 |  |  |
| PLWHA (3) | |  | 17 | 9 | 2 | 8 |  | 1 |  |  |  |  |  |  | 2 |  | 1 |
| General population (1) | |  | 27 | 2 | 4 | 5 |  | 1 |  |  |  |  |  |  |  |  | 5 |
| **Clients of FSWs** | |  |  |  |  |  |  |  |  |  |  |  |  |  |  |  |  |
| Policy makers (1) | |  | 3 | 13 |  |  |  | 6 |  |  |  |  |  |  |  | 1 |  |
| Health care workers (2) | |  | 9 | 15 |  | 4 |  | 5 | 1 | 1 |  |  |  |  | 1 |  | 2 |
| PLWHA (4) | |  | 9 | 10 |  | 3 |  | 3 |  |  |  |  |  | 1 | 1 |  | 6 |
| General population (1) | |  | 19 | 4 | 3 | 3 |  | 1 |  |  |  |  | 3 | 1 | 2 |  | 7 |
| **MSM** | |  |  |  |  |  |  |  |  |  |  |  |  |  |  |  |  |
| Policy makers (1) | |  | 5 | 1 | 1 | 2 |  | 5 |  | 1 | 1 | 2 |  |  | 2 |  | 2 |
| Health care workers (4) | |  | 12 | 2 | 4 | 3 |  | 6 |  | 2 | 2 | 1 |  |  | 2 | 1 | 2 |
| PLWHA (6) | |  | 13 | 4 | 1 | 5 |  | 1 |  | 1 | 1 |  |  |  | 1 |  | 7 |
| General population (4) | |  | 11 | 1 | 1 | 2 |  | 4 |  |  |  | 11 | 1 | 2 |  |  | 3 |
| **Transgender** | |  |  |  |  |  |  |  |  |  |  |  |  |  |  |  |  |
| Policy makers (1) | |  | 4 | 3 |  | 3 | 5 |  |  | 2 | 1 | 1 |  |  | 1 | 1 |  |
| Health care workers (7) | |  | 11 | 9 | 3 | 2 |  | 3 | 1 | 1 |  | 2 |  |  | 2 |  |  |
| PLWHA (6) | |  | 14 | 3 |  | 6 |  |  | 4 | 1 |  | 3 |  |  | 2 |  | 3 |
| General population (3) | |  | 12 |  | 2 | 7 | 2 |  |  | 3 | 1 | 8 |  | 1 |  |  | 2 |
| **Prisoners** |  | |  |  |  |  |  |  |  |  |  |  |  |  |  |  |  |
| Policy makers (2) |  | | 6 | 3 |  | 3 | 1 | 3 | 1 |  | 1 |  |  |  |  |  | 1 |
| Health care workers (6) |  | | 22 | 11 |  | 1 | 2 | 1 |  | 1 |  |  | 1 |  | 1 |  |  |
| PLWHA (4) |  | | 18 | 3 |  | 9 | 6 | 2 | 1 | 3 |  |  |  | 1 | 2 |  |  |
| General population (3) |  | | 13 | 1 | 2 | 4 | 3 | 2 |  | 11 |  |  |  | 2 |  |  |  |
| Table 3 (continued) | | | | | | | | | | | | | | | | | |
|  |  | | Reasons to prioritize risk group (Likert scores 3-5) | | | | | |  | Reasons to not prioritize group (Likert scores 1-3) | | | | | | | |
|  |  | | At high  HIV risk | Important in spread of HIV epidemic | Large number of people (infected) | Equity: less access to services, more vulnerable | Feasibility: effective interventions are available/ easy group to reach | Feasibility: no effective interventions available/ difficult group to target | Other | At low HIV risk | Not important in spread of HIV epidemic | Small number of people (infected) | Equity: group with relatively high socio economic status | Equity: own responsibility | Feasibility: effective interventions are available/ group is already targeted | Feasibility: no effective interventions available/ difficult group to target | No need for services (already show safe behaviour) |
| **Partners of HIV+ people** |  | |  |  |  |  |  |  |  |  |  |  |  |  |  |  |  |
| Policy makers (1) |  | | 9 | 2 |  |  | 5 | 5 |  |  |  |  |  |  |  |  |  |
| Health care workers (2) |  | | 25 | 3 |  | 7 | 2 |  |  | 1 |  |  |  |  |  | 3 |  |
| PLWHA (5) |  | | 27 | 4 | 1 | 10 | 1 |  | 1 |  |  |  |  |  | 2 |  | 1 |
| General population (1) |  | | 27 | 2 | 2 | 3 | 1 |  |  |  |  | 1 |  | 1 |  |  | 4 |
| **People at low risk** |  | |  |  |  |  |  |  |  |  |  |  |  |  |  |  |  |
| Policy makers (1) |  | | 9 | 1 |  |  | 2 | 3 |  | 3 |  |  |  |  | 1 | 1 | 1 |
| Health care workers (7) |  | |  |  | 3 | 2 |  | 2 | 1 | 18 |  |  |  |  | 5 |  |  |
| PLWHA (7) |  | | 3 | 1 | 1 | 12 |  |  |  | 20 |  |  |  |  | 1 |  | 2 |
| General population (4) |  | |  |  | 3 | 3 | 5 |  |  | 15 |  |  |  |  | 1 |  | 5 |
| **Total** |  | |  |  |  |  |  |  |  |  |  |  |  |  |  |  |  |
| Policy makers (9) |  | | 42 | 42 | 6 | 12 | 16 | 26 | 1 | 6 | 4 | 4 | 0 | 0 | 4 | 3 | 4 |
| Health care workers (31) |  | | 110 | 70 | 23 | 21 | 4 | 27 | 3 | 24 | 2 | 3 | 1 | 0 | 15 | 4 | 4 |
| PLWHA (35) |  | | 121 | 43 | 9 | 57 | 7 | 11 | 7 | 25 | 1 | 3 | 0 | 3 | 11 | 1 | 20 |
| General population (17) |  | | 132 | 13 | 22 | 28 | 11 | 13 | 0 | 29 | 1 | 21 | 5 | 8 | 3 | 0 | 29 |
| **All stakeholders** |  | | **405** | **168** | **60** | **118** | **38** | **77** | **11** | **84** | **8** | **31** | **6** | **11** | **33** | **8** | **57** |
| IDUs = injecting drug users, PLWHA = people living with HIV/AIDS, MSM = men having sex with men, FSW = female sex workers | | | | | | | | | | | | | | | | | |
